# Specific lysophosphatidylcholine and acylcarnitine related to sarcopenia and its components in older men

**DOI:** 10.1186/s12877-022-02953-4

**Published:** 2022-03-25

**Authors:** Li Meng, Ruiyue Yang, Daguang Wang, Wenbin Wu, Jing Shi, Ji Shen, Yamin Dang, Guoqing Fan, Hong Shi, Jun Dong, Huan Xi, Pulin Yu

**Affiliations:** 1 Department of Geriatrics, National Clinical Research Center for Geriatrics, Beijing Hospital, National Center of Gerontology, Institute of Geriatric Medicine, Chinese Academy of Medical Sciences, 100730 Beijing, People’s Republic of China; 2 The Key Laboratory of Geriatrics, Beijing Institute of Geriatrics, Institute of Geriatric Medicine, Chinese Academy of Medical Sciences, Beijing Hospital/National Center of Gerontology of National Health Commission, 100730 Beijing, People’s Republic of China; 3 Department of Laboratory Medicine, Beijing Hospital, National Center of Gerontology, Institute of Geriatric Medicine, Chinese Academy of Medical Sciences, 100730 Beijing, People’s Republic of China

**Keywords:** Sarcopenia, Muscle, Metabolomics, Biomarkers, Multivariate analyses, Older men

## Abstract

**Background:**

Metabolic profiling may provide insights into the pathogenesis and identification of sarcopenia; however, data on the metabolic basis of sarcopenia and muscle-related parameters among older adults remain incompletely understood. This study aimed to identify the associations of metabolites with sarcopenia and its components, and to explore metabolic perturbations in older men, who have a higher prevalence of sarcopenia than women.

**Methods:**

We simultaneously measured the concentrations of amino acids, carnitine, acylcarnitines, and lysophosphatidylcholines (LPCs) in serum samples from a cross-sectional study of 246 Chinese older men, using targeted metabolomics. Sarcopenia and its components, including skeletal muscle index (SMI), 6-m gait speed, and handgrip strength were assessed according to the algorithm of the Asian Working Group for Sarcopenia criteria. Associations were determined by univariate and multivariate analyses.

**Results:**

Sixty-five (26.4%) older men with sarcopenia and 181 (73.6%) without sarcopenia were included in the study. The level of isovalerylcarnitine (C5) was associated with the presence of sarcopenia and SMI. Regarding the overlapped metabolites for muscle parameters, among ten metabolites associated with muscle mass, six metabolites including leucine, octanoyl-L-carnitine (C8), decanoyl-L-carnitine (C10), dodecanoyl-L-carnitine (C12) and tetradecanoyl-L-carnitine (C14), and LPC18:2 were associated with handgrip strength, and three of which (C12, C14, and LPC18:2) were also associated with gait speed. Specifically, tryptophan was positively associated and glycine was negatively associated with handgrip strength, while glutamate was positively correlated with gait speed. Isoleucine, branched chain amino acids, and LPC16:0 were positively associated with SMI. Moreover, the levels of LPC 16:0,18:2 and 18:0 contributed significantly to the model discriminating between older men with and without sarcopenia, whereas there were no significant associations for other amino acids, acylcarnitines, and LPC lipids.

**Conclusions:**

These results showed that specific and overlapped metabolites are associated with sarcopenic parameters in older men. This study highlights the potential roles of acylcarnitines and LPCs in sarcopenia and its components, which may provide valuable information regarding the pathogenesis and management of sarcopenia.

**Supplementary Information:**

The online version contains supplementary material available at 10.1186/s12877-022-02953-4.

## Introduction

Sarcopenia is a prototypical geriatric condition characterized by age-associated progressive loss of skeletal muscle mass, coupled with decreased muscle strength and function. The prevalence of sarcopenia ranges from 9.9%–40.4% in community-dwelling older adults [1] and 5%–13% in people aged 60–70 years, but the proportion increases to 11%–50% in people aged > 80 years [2]. Sarcopenia can contribute significantly to the risk of frailty, the progression of disability, poor quality of life, and mortality among the older adults. Sarcopenia has thus become a “blockbuster” condition in geriatrics, given its increasing prevalence in a globally aging world [3–5].

Previous studies have analyzed the pathological basis of sarcopenia and its associated factors, but a definitive pathophysiological framework of these conditions is still lacking, and potential biomarkers contributing to this condition remain largely unknown [2]. Considering the multidimensionality of sarcopenia, a single-biomarker approach cannot explain the biological foundations of this condition, and a more comprehensive approach is needed to study this age-related condition. Metabolomic analysis has emerged as a powerful technique, providing an interface between biological pathways and clinical manifestations of disease conditions [6], with the potential to identify metabolic factors that contribute to sarcopenia, and thus allowing the early identification and management of this condition [7]. Compared with non-targeted metabolomics, targeted metabolomics allows for more accurate quantification to detect specific metabolites. Many investigations have used targeted metabolomics for identification of metabolic change correlated with sarcopenia or frailty, while non-targeted metabolomics may be more widely used in screening frailty-related metabolic biomarkers [8, 9]. However, the metabolites associated with sarcopenia and muscle-related parameters have not been comprehensively profiled and examined.

Specific patterns of amino acids have been associated with sarcopenia, but previous studies have generated conflicting results [10–12]. Additionally, a few studies examining the roles of lipid metabolism, including carnitines, acylcarnitines, and lysophosphatidylcholine (LPC) species, have shown the associations with insulin resistance or inflammation [13–15]. These metabolites are also involved in mitochondrial function and redox homeostasis, which may contribute to the mechanisms of frailty or sarcopenia [16–18]. However, relatively less research has been carried out in the area of relationship between these lipid metabolites with sarcopenia and its components.. It is therefore important to carry out extensive measurements of these metabolic species to further our insights into the metabolic basis of sarcopenia.

Comprehensive metabolomic studies of muscle mass, muscle strength, and physical function are lacking, and few studies have considered these sarcopenic muscle-related simultaneously. However, recent evidence has suggested that the age-related decline in muscle strength is greater than that expected based on the decline in muscle mass alone, and decreased muscle strength is a much better predictor of functional limitations and poor health in older adults than muscle mass alone [19]. Recent Asian definitions of sarcopenia have incorporated both loss of muscle mass and muscle strength or function [20, 21], and European working group on sarcopenia in older people(EWGSOP) guidance also recommend the importance of muscle strength over muscle mass [22], highlighting the importance of considering the different components of sarcopenia for obtaining an understanding of its complex biological mechanism. Some investigations of the metabolic correlations of amino acids and LPC with sarcopenic components has been linked to muscle mass, gait speed and chair stand test, but little research has focused on the associations of acylcarnitines with gait speed, or LPC with handgrip strength in older adults [23–27]. In the Health, Aging and Body Composition (ABC) study, the metabolites most strongly correlated with appendicular skeletal muscle mass included isovalerylcarnitine (C5) and branched-chain amino acids (BCAAs) [26]. Baseline plasma concentration of LPC 18:2 was an independent predictor of the rate of change of gait speed in the Baltimore Longitudinal Study of Aging [27]. There is evidence that the levels of aspartic acid and glutamic acid might make an essential contribution to regulate muscle mass and strength [28]. Lower blood levels of essential amino acids (EAA), BCAAs, especially leucine, were also found to be associated with lower skeletal muscle index (SMI), strength, and longer time to complete the chair stand test [29]. However, one previous study showed that there were higher blood levels of isoleucine, leucine, tryptophan, serotonin, and methionine in the participants with low muscle quality compared to those with high muscle quality, which might be the result of impaired metabolism of amino acids, and reduced uptake of skeletal muscle [25]. Moreover, data on the associations of metabolites with sarcopenia and its components among older Chinese older men, who have a higher prevalence of sarcopenia than women, are scarce [21, 30, 31] compared with related studies mainly involved North American and European people. In this study, we simultaneously explored several metabolic classes in terms of their relationships to sarcopenia and muscle parameters (components or traits). We used our established targeted metabolomics approach and multivariate analysis to identify specific and overlapped metabolic factors for these key sarcopenia traits in a cross-sectional study of Chinese men. The overall goal of this exploratory study was to explore different metabolites associated with muscle mass, strength, and function, and to provide valuable information relating to the pathogenesis and management of sarcopenia.

## Materials and methods

### Clinical data of participants

This cross-sectional study recruited participants attending the Geriatric Medicine Department of Beijing Hospital for physical examination between August 2016 and May 2018. Older Chinese men aged 62–100 years (*n* = 246) with available data for sarcopenia identification were included. Demographic variables included age, body mass index (BMI), smoking status, and frequency of alcohol drinking, and the participants’ clinical characteristics were assessed for health status by comprehensive geriatric assessment: Physical activity was evaluated using the Chinese version of the International Physical Activity Questionnaire [32];Malnutrition risk was evaluated using the Mini-Nutritional Assessment Short-Form (MNA-SF), ≥ 12scores as normal, ≤ 11 scores as possible malnutrition [33]; Polypharmacy was defined as the use of more than five medications [34]; cognitive (Mini-Mental State Examination) and psychological functions (Geriatric Depression Scale). Participants were assessed for disability in six activities of daily living(ADL) tasks (Walking, dressing, bathing, eating, brushing tooth or comb hair, and toileting) and eight instrumental ADL (IADL) tasks (preparing hot meals, doing household chores, Use of public vehicles, washing clothes,shopping, Managing money, Making telephone calls, and taking medications), < 16 scores as normal, ≥ 16 scores as disability (ADL decline) to some degree [35]. Overnight fasting blood samples were collected and serum was isolated and stored at − 80 °C until further analysis. Routine biochemical parameters were evaluated using an automated analyzer. The inclusion criteria were men older than 60 years, ability to understand and complete the questionnaires, and not suffering from severe mental or cognitive disorders. Subjects with malignant tumors, blood system diseases, chronic obstructive pulmonary disease, autoimmune diseases, infectious diseases, and subjects taking amino acid supplements were excluded. The study was approved by the Ethics Committee of the Beijing Hospital of the National Health Commission, and all participants provided written informed consent.

### Sarcopenia and muscle parameters

Sarcopenia was defined according to the algorithm of the Asian Working Group for Sarcopenia (AWGS) criteria released in 2019 [21], including poor muscle mass and low muscle strength and/or low physical performance. Maximum handgrip strength of either hand was assessed twice using digital dynamometers (WCS-II, Beijing) to assess low muscle strength. Low physical performance was evaluated using the average of two timed walk tests over a 6-m course. Appendicular skeletal muscle mass (ASM) was measured using a bioelectrical impedance data acquisition system (Inbody720; Biospace Co, Ltd, Seoul, Korea). Low muscle strength was defined as handgrip strength < 28 kg for men and low physical performance was defined as 6-m walk speed < 1.0 m/s. The cutoff for height-adjusted muscle mass (i.e. skeletal muscle mass index [SMI]) by bioelectrical impedance was < 7.0 kg/m^2^ in men. Physical function was assessed using the timed up and go test (TUG) [32], five times sit-to-stand test (5STS) [36], and balance [37].

### Measurements of serum metabolites

Serum metabolites were measured by targeted liquid chromatography-tandem mass spectrometry (LC–MS/MS), as described previously [38, 39]. The measured metabolites included branched-chain and aromatic amino acids (leucine, isoleucine, valine, phenylalanine, tryptophan, and tyrosine), glutamate, glutamine, glutamine/glutamate ratio, and carnitine, several species of acylcarnitines, and LPCs levels (16:0, 18:0, 18:1, and 18:2, trimethylamine oxide, betaine).

Briefly, aliquots of 0.01 mL of calibrators or serum samples were mixed with 0.01 mL of isotopically-labeled internal standards, followed by the addition of 1 mL of isopropanol. After vortexing and centrifuging (3500 rpm), 0.2 mL of the supernatant was transferred and analyzed by LC–MS/MS using an AB Sciex 5500 QTRAP tandem mass spectrometer (Framingham, MA, USA) equipped with an Agilent 1260 Series HPLC system (Santa Clara, CA, USA).

### Statistical analysis

Continuous variables were expressed as the mean ± standard deviation for normally distributed data and the median (P25, P75) for non-normally distributed variables. Categorical data were expressed as percentages. The normal distribution of the data was ascertained by the Kolmogorov–Smirnov test. Comparisons between sarcopenia and nonsarcopenia were analyzed by independent *t*-tests and χ^2^ tests or Fisher’s exact tests as appropriate. Spearman’s correlation analysis was used to examine the relationships of metabolite concentrations with gait speed, SMI, and handgrip strength. All statistical tests were two-tailed and a p-value < 0.05 was considered to be statistically significant.

Linear regression (SAS JMP 13.0.0, Cary, NC, USA) was used in multivariable analyses to determine the associations between individual metabolites and muscle parameters.The clinical potential confounding covariates, that were significantly associated with sarcopenia or its components in the above univariate analyses and previous studies  [12, 40, 41] were adjusted in two models, as follows: Model 1: age, body mass index, smoking status, alcohol drinking, malnutrition and physical activity, Model 2: further controlled for polypharmacy and comorbidities, to ensure that the detected associations were independent of clinical factors.We also carried out multivariable-adjusted logistic regression analysis to screen the significant factors that influenced sarcopenia. Logistic regressions (SAS 9.3 Institute Inc., Cary, NC, USA) were performed to estimate the associations between metabolites and the presence of sarcopenia, gradually adjusting for potential confounding by model 1(age and smoking status, BMI, malnutrition, physical activity, cognition), and model 2 (added by hemoglobin, red blood cells, albumin). Polypharmacy and comorbidities did not enter the regression equation. Level of significance was set at *p* < 0.05.

To enhance the reliability of the important factors for sarcopenia, orthogonal partial least squares discriminant analysis (OPLS-DA) was preliminarily applied to distinguish those metabolites related to sarcopenia and to enhance the separation between groups using rotating principal component analysis. This multivariate analyses were performed using SIMCA-P v.14.1, as described previously [42, 43], because of its versatility and ability to deal with highly correlated predictors and it is more effective in focusing the correlated information onto the first predictive component instead of scattering it onto the subsequent components [44]. The quality of OPLS-DA model was validated by the goodness of fit (R2) and the goodness of prediction indicated by the cumulated Q2 value (Q2 cum). Permutation tests were used to assess whether the model was overfitted. The area under the receiver operating characteristic (ROC) curve was used as an indicator of discriminatory power. Important factors were identified by S-Plot and variable importance for the projection (VIP) score. A VIP score > 1 indicated that the independent variable was an important factor for distinguishing sarcopenia status. Metabolites with a VIP > 1 were further subjected to univariate analysis to measure the significance.

## Results

### Clinical characteristics of participants with and without sarcopenia

The study participants included 65 (26.4%) sarcopenia and 181 (73.6%) nonsarcopenia. Among the sarcopenic men, 52.8% had low handgrip strength, and 70.8% had slower gait speed. Moreover, not all the older sarcopenic men in this study met all three criteria, and 30.7% of men had decreased gait speed and 17.7% had low handgrip strength in nonsarcopenia (*P* = 0.000).

The clinical characteristics of sarcopenia and nonsarcopenia are shown in Table 1. Compared with nonsarcopenic group, the age was not significant older in sarcopenic group. The sarcopenic older men had a lower activity of daily living than the nonsarcopenic group. There were no differences in nutrition status and physical function (TUG, 5STS and balance) between the two groups. Men with sarcopenia had lower levels of red blood cells, hemoglobin, and albumin. However, there were no significant differences in comorbidities, polypharmacy, cognitive decline, depression status, and other general laboratory parameters between the two groups.

**Table 1:** Clinical characteristics of the study participants stratified by sarcopenia status

Variables	Nonsarcopenia(*n* = 181)	Sarcopenia(*n* = 65)	t/χ^2^/Z	*p*-value
Age(years)^a^	78.6 ± 7.4	80.9 ± 8.5	1.898	0.058
Current smoking	20.8%	12.5%	2.406	0.1210
Alcohol intake	17.2%	11.1%	1.474	0.225
BMI(kg/m^2^)^b^	25.8(23.7, 27.4)	23.0(20.9, 24.9)	-5.877	**0.000**
Malnutrition	26.04%	33.33%	1.380	0.240
Comorbidities^a^	3.6 ± 1.5	4.7 ± 1.9	-0.801	0.423
Polypharmacy	33.0%	43.8%	2.23	0.134
ADL decline	6.8%	23.6%	17.002	**0.0001**
Cognitive decline	10.4%	8.3%	0.256	0.613
Depression	2.6%	6.9%	5.952	0.114
Lower activity	18.8%	20.8%	0.146	0.703
**Physical function**				
Poor balance	85%	86%	0.380	0.538
5-time sit to stand test ≥ 10 s	85%	86%	0.377	0.539
Time up and go test, ≥ 12 s	84%	86%	0.384	0.535
**Muscle-related parameters**				
ASM(kg)^b^	21.3(18.9, 23.1)	17.0(12.1, 18.5)	-7.388	**0.000**
SMI(kg/m^2^) ^b^	7.4(7.0, 87.9)	6.3(4.7, 6.7)	-8.179	**0.000**
Handgrip strength (kg) ^b^	30.6(20.1, 35.1)	17.0(0.0, 24.6)	-6.699	**0.000**
Gait speed (m/s) ^b^	1.0(0.5, 1.0)	0.7(0.5, 1.0)	-2.818	**0.005**
**Blood general parameters**^b^				
Red blood cell (10^12^/L)	4.62(4.36, 4.96)	4.52(4.23, 4.84)	-1.998	**0.046**
White blood cell (10^9^/L)	5.9(4.8, 7.0)	5.8(4.7, 7.0)	-0.240	0.810
Platelets (10^9^/L)	182.0(156.5, 210.5)	192.0(162.5, 220.0)	1.519	0.129
Hemoglobin (g/L)	143(134, 151)	137(127, 145)	-3.630	**0.000**
Fasting glucose (mmol/L)	5.7(5.3, 6.4)	5.7(5.3, 6.2)	-0.473	0.636
HbALc(%)	57(5.4, 6.1)	5.8(5.5, 6.2)	1.039	0.299
Triglyceride (mmol/L)	1.3(0.9, 1.8)	1.1(0.8, 1.5)	-1.620	0.105
Total cholesterol (mmol/L)	4.0(3.3, 4.9)	4.1(3.4, 4.7)	0.291	0.771
LDL-C(mmol/L)	2.3(1.8, 3.0)	2.3(1.9, 2.8)	0.018	0.986
HDL-C(mmol/L)	1.2(1.0, 1.4)	1.2(1.0, 14)	0.189	0.850
ALT(U/L)	17.8(14.5, 23.0)	18(14.5, 23.5)	-0.236	0..814
AST(U/L)	27(23, 31.5)	27(24, 32)	0.552	0.581
Albumin (g/L)	41.0(0.0, 43.0)	39.5(0.0, 42.0)	-2.536	**0.011**
Uric acid (µmoo/L)	349.0(309.0, 417.5)	339.5(275.0, 407.0)	-1.582	0.114
Creatinine (µmoo/L)	77.0(68.0, 91.0)	76.0(62.0, 86.5)	-0.1.274	0.203
BUN (mmoo/L)	5.6(4.5, 6.9)	5.8(5.1, 6.7)	1.202	0229

^a^Data are shown as Mean ± standard deviation, ^b^shown as medians and 25^th^-75^th^ percentiles. Abbreviates: *ADL* activities of daily living, *BMI* body mass index, *ASM* appendicular skeletal muscle, *SMI* skeletal muscle index, *HbALc* Glycosylated Hemoglobin, *LDL-C* low density lipoprotein cholesterol, *HDL-C* high density lipoprotein cholesterol, *ALT* glutamate pyruvate transaminase, *AST* Aspartate aminotransferase, *BUN* Blood urea nitrogen

### Spearman’s correlations between serum metabolites and muscle parameters

Spearman’s analysis showed significant correlations between metabolites and muscle traits (Table 2). Although several metabolites were significantly correlated with muscle parameters, most correlation coefficients were low.

**Table 2:** Significant Spearman correlations of serum metabolite with muscle parameters

Variables		Ileucine	Leucine	BCAA	Glu	Tryptophan	Glycine	C5	C6	C8	C10	C12	C14	LPC 18:2
SMI	*r*	0.176	0.135	0.141	0.054	0.076	-0.086	0.190	-0.108	-0.156	-0.145	-0.159	-0.158	0.140
	*P*	**0.004**	**0.028**	**0.022**	0.382	0.218	0.169	**0.0267**	0.081	**0.011**	**0.019**	**0.010**	**0.010**	**0.023**
Handgrip strength	*r*	0.105	0.125	0.115	0.097	0.235	-0.136	-0.088	-0.155	-0.190	-0.188	-0.206	-0.188	0.130
	*P*	0.090	**0.042**	0.063	0.117	**0.000**	**0.027**	0.157	**0.012**	**0.002**	**0.002**	**0.001**	**0.002**	**0.005**
Gait speed	*r*	0.078	0.078	0.075	0.146	0.120	-0.019	-0.107	-0.031	-0.027	-0.028	-0.050	-0.095	0.171
	*P*	0.206	0.206	0.228	**0.017**	0.051	0.763	0.083	0.622	0.661	0.656	-0.206	0.123	**0.035**

Abbreviates: SMI: skeletal muscle index, BCAA: branched chain amino acids, Glu: Glutamte, C5:valeryl-L-carnitine,C6:AcyCNhexenoyl-L-carnitine,C8:octanoyl-L-carnitine,C10:decanoyl-L-carnitin, C12:dodecanoy-L-carnitine,C14:tetradecanoyl-L-car-nitine, LPC18:2: 1-linoleoyl-2-hydroxy-sn-glycero-3-phosphocholine

SMI was positively correlated with isoleucine, leucine, BCAAs, C5, and LPC 18:2, and negatively correlated with octanoyl-L-carnitine (C8),decanoyl-L-carnitine (C10), dodecanoyl-L-carnitine (C12) and tetradecanoyl-L-carnitine (C14). Levels of leucine, tryptophan, and LPC 18:2 metabolites were positively associated with handgrip strength, whereas glycine, AcyCNhexenoyl-L-carnitine(C6), C8, C10, decenoyl-L-carnitine(C10:1), C12, and C14 were negatively related to handgrip strength. Glutamic acid and LPC 18:2 were correlated with gait speed. There were no significant relationship between carnitine metabolites and gait speed.

### Metabolites associated with muscle parameters by linear regression analysis

Linear regression models were used to analyze the independent associations of identified metabolic factors with the three components of sarcopenia, after adjustments for age, BMI, smoking, alcohol drinking, physical activity, malnutrition, polypharmacy, and comorbidities (Table 3 and supplymentary Table 1, 2, 3 ).

**Table 3:** Liner regression analysis for associations of serum metabolites with muscle parameters

variables	Handgrip strength			Gait speed			SMI		
	*β*	*SE*	*P*	*β*	*SE*	*P*	*β*	*SE*	*P*
Valine	-0.030	0.062	0.627	-0.001	0.004	0.767	-0.009	0.015	0.526
Ileucine	-0.088	0.168	0.599	-0.001	0.011	0.929	-0.010	0.040	0.807
Leucine	0.0125	0.098	0.898	0.002	0.007	0.706	-0.003	0.023	0.897
Tyrosine	0.1364	0.130	0.294	0.005	0.009	0.598	-0.033	0.0318	0.280
Tryptophan	0.362	0.132	**0.007**	0.016	0.009	0.069	-0.021	0.032	0.509
Glutamine	0.009	0.056	0.876	-0.002	0.003	0.675	-0.013	0.013	0.316
Glutamate	0.041	0.079	0.604	0.010	0.005	**0.049**	0.004	0.019	0.832
Glycine	-0.039	0.019	**0.043**	-0.002	0.001	0.156	-0.006	0.005	0.171
Gln/Glu	-0.555	0.852	0.516	-0.091	0.057	0.113	-0.156	0.202	0.441
BCAA	-0.011	0.033	0.751	-0.000	0.002	0.962	-0.003	0.008	0.668
C2	-0.473	0.547	0.388	-0.070	0.037	0.058	-0.047	0.129	0.714
C5	18.866	46.553	0.686	1.126	3.140	0.720	15.414	10.965	0.161
C8	-14.478	8.900	0.105	-0.400	0.603	0.508	0.437	2.116	0.837
C10	-12.446	7.014	0.077	-0.434	0.475	0.363	0.045	1.669	0.979
C12	-61.396	29.987	**0.042**	-4.337	2.021	**0.033**	-1.499	7.151	0.834
C14	-228.4	92.431	**0.014**	-16.739	6.219	**0.008**	-19.489	22.096	0.379
C16	-8.973	32.181	0.781	-2.307	2.166	0.288	-1.484	7.609	0.846
LPC16:0	0.003	0.013	0.818	0.002	0.001	0.080	0.006	0.003	**0.044**
LPC18:2	0.061	0.036	0.090	0.004	0.002	0.131	0.012	0.008	0.148

Adjusted variables: Age, BMI, smoking, alcohol drinking, physical activity, malnutrition, polypharmacy, comorbidities

Abbreviates: *SMI* skeletal muscle index, *Glu* glutamate, *Gln* glutamine, *BCAA* branched-chain-amino acids, *C2* acetylcarnitine, C5:valeryl-L-carnitine,C8:octanoyl-L-carnitine,C10:decanoyl-L-carnitin,C12:dodecanoy-L-carnitine,C14:tetradecanoyl-L-car-nitine,C16:hexadec-C16anoyl-L-carnitine, LPC16:0:1-palmitoyl-2-hydroxy-sn-glycero-3-phosphocholine, LPC18:2: 1-linoleoyl-2-hydroxy-sn-glycero-3-phosphocholine

There were significant associations between metabolites and gait speed, including positive associations for glutamate and negative associations for two acylcarnitines (C12 and C14). However, the correlation between short-chain acylcarnitine (C2) and gait speed was no longer statistically significant after controlling for comorbidities and polypharmacy, and there were no significant associations between lysophospholipids and gait speed.

Regarding handgrip strength, tryptophan was positively, and glycine, the acylcarnitines C12 and C14 were negatively associated with handgrip strength, after adjustments for potential confounders. There were no significant associations between lysophospholipids and handgrip strength.

With respect to muscle mass, only LPC 16:0 was significantly associated with SMI after controlling for all the covariates. Regarding acylcarnitine, the levels of C12 and C14 were negatively associated with both reduced handgrip strength and slow gait speed in apart from SMI. Additionally, no effect of BCAAs on the three components of sarcopenia was found in linear regression analysis.

### Identification of metabolites associated with sarcopenia by multivariate analyses

Logistic regression analyses were performed to investigate the associations between metabolic factors and the presence of sarcopenia. Sarcopenia was significantly associated with C5 as a categorical variable (third compared with lowest quartile) (odds ratio (95% confidence interval: 0.363 (0.153, 0.865), *p* = 0.0222) after adjusting for age, BMI, smoking status, malnutrition, cognition, and hemoglobin; however, the association was no longer significant after adjusting for the covariates of serum albumin, red blood cells. There were no significant associations between amino acids, other acylcarnitines, and LPC and the presence of sarcopenia in Logistic regression model.

**Fig. 1:**
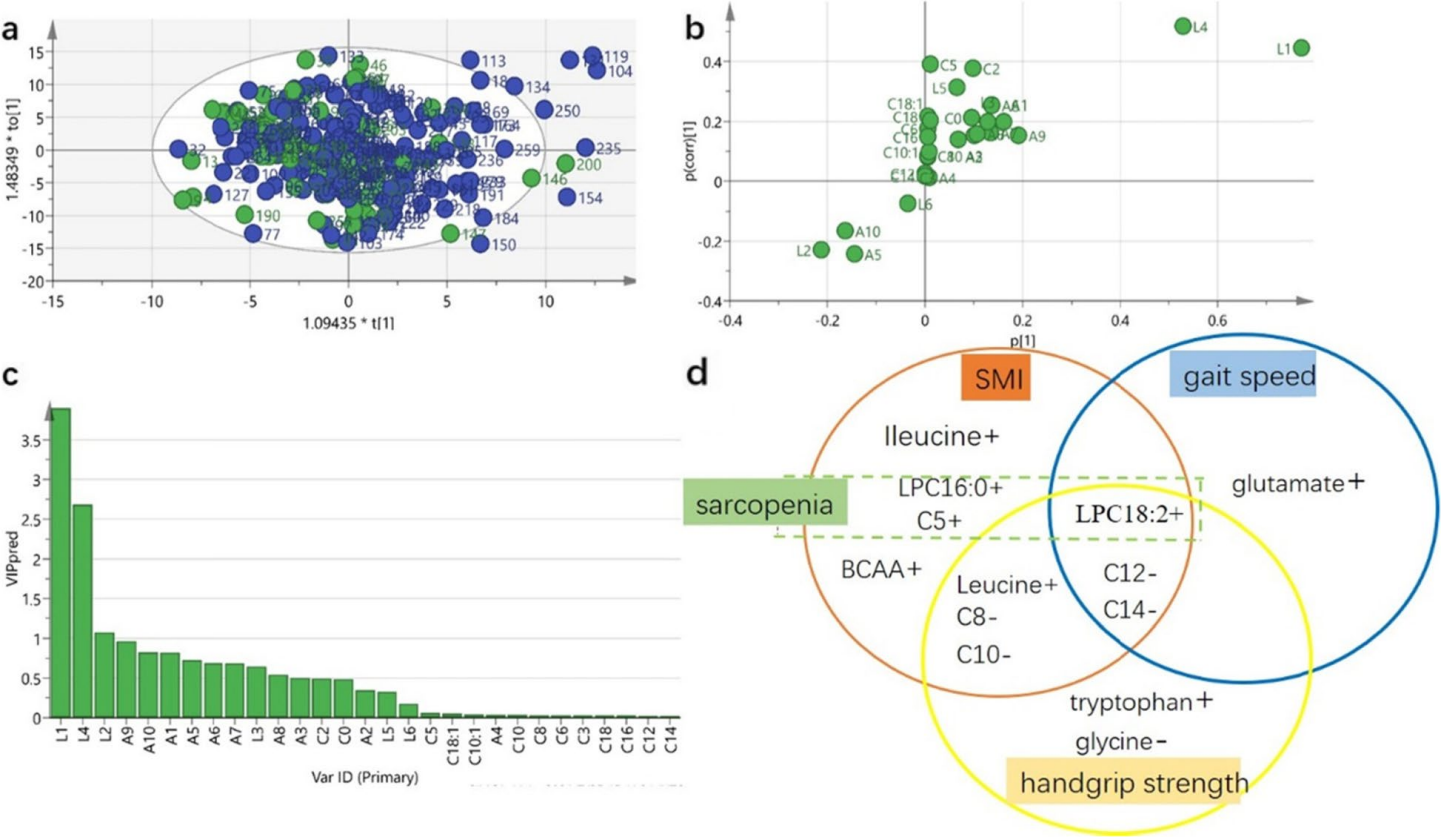
Orthogonal partial least squares discriminant analysis and summary of metabolites for sarcopenia and its components. (a)Scatter plot of OPLS-DA model analysis for sarcopenia (green circle) versus nonsarcopenia (blue circle) by the main components. (b)s-plot of OPLS-DA model of sarcopenia and nonsarcopenia. (c)Ordered list of metabolites with higher discrimination ability based on variable importance for the projection (VIP) score for separation of sarcopenia and nonsarcopenia.(d)Specific and overlapped metabolite for muscle parameters and sarcopenia(+ : positive association, -:negative association) Abbreviates:L1:1-palmitoyl-2-hydroxy-sn-glycero-3-phosphocholine,L4:1-linoleoyl-2-hydroxy-sn-glycero-3-phosphocholine,L2:1-stearoyl-2-hydroxy-sn-glycero-3-phosphocholine,L3:1-oleoyl-2-hydroxy-sn-glycero-3-phosphocholine,A9:glycine,A10:alanine,A1:valine,A5:phenylalanine A6:tryptophan,A7:glutamine,A3:leucine,A4:tyrosine,A2:Ileucine,A8:glutamate,C0:carnitine, C2:acetylcarnitine,C3:propionyl-L-carnitine,C5:valeryl-L-carnitine,C6:AcyCNhexenoyl-L-carnitine,C8:octanoyl-L-carnitine,C10:decanoyl-L-carnitin,C10:1:decenoyl-L-carnitine,C12:dodecanoy-L-carnitine,C14:tetradecanoyl-L-car-nitine,C16:hexadec-C16anoyl-L-carnitine,C18:octadecanoyl-L-carnitine,C18:1:octadecenoyl-L-carnitine

To verify the different metabolic features in participants with sarcopenia, we constructed an OPLS-DA classification model to select characteristic metabolites that distinguished sarcopenia from non-sarcopenia along the main component. The OPLS-DA model revealed no clear separation between older sarcopenic and non-sarcopenic participants (Fig. 1A). The parameters of goodness of fit and goodness of prediction in the preliminary OPLS-DA model showed that this model had a certain ability to explain but low ability to predict sarcopenia (R2X = 0.615, R2Y = 0.172, Q2 = 0.0316). The S-Plot (Fig. 1B) and VIP score (Fig. 1C) showed the possible important metabolic discriminants between the two groups. The variables corresponding to VIP are reported in Table 4. Three LPCs contributed significantly to the discrimination model: participants with sarcopenia were characterized by lower levels of LPC16:0, LPC18:2, and LPC18:0. After univariate analysis, sarcopenic participants had significantly lower level of C5 compared with the non-sarcopenic group (*P* = 0.026), while serum concentrations of other amino acids, acylcarnitines, and LPC lipid were similar between the groups. Permutation tests indicated that the model was reliable and had a low risk of over-fitting. The area under the curve (AUC) of the model was greater than 0.7 with the discriminatory power to some accuracy (Supplementary Fig. 1).

**Table 4:** Serum concentrations of discriminant analytes and variable importance in projection (VIP) values in participants with and without sarcopenia

variables	Nonsarcopenia	Sarcopenia	VIP	t/Z	P value
	(*n* = 181)	(*n* = 65)			
LPC16:0	117.27(88.63,142.54)	105.16(85.23,129.62)	**3.896**	-1.543	0.123
LPC18:2	28.67(19.88,36.35)	24.6(18,34.32)	**2.681**	-1.631	0.103
LPC18:0	36.78(30.52,42.05)	36.01(30.56,43.05)	**1.077**	0.204	0.839
Glycine	38.66(32.64,48.82)	39.31(35.04,43.34)	0.966	0.0580	0.954
Alanine	49.19(41.52,57.65)	49.54(42.41,57.35)	0.825	0.053	0.958
Valine	40.07(34.88,44.54)	38.19(33.88,42.16)	0.820	-1.566	0.1174
Phenylalanine	18.71(16.48,21.84)	19.09(17.01,22.67)	0.728	1.011	0.312
Tryptophan	16.8(14.88,19.15)	16.16(14.64,18.35)	0.688	-1.530	0.126
Glutamine	25.32(20.28,30.74)	26.75(20.64,29.74)	0.683	-0.380	0.704
LPC18:1	17.5(14.87,20.82)	16.91(14.73,20.19)	0.645	-0.607	0.544
Glutamate	21.93(19.23,24.89)	20.97(17.09,24.14)	0.543	-1.608	0.108
Leucine	25.54(22.41,28.9)	25.43(21.24,27.66)	0.504	-1.059	0.290
C2	2.16(1.69,2.58)	2.05(1.61,2.59)	0.496	-0.775	0.439
Carnitine	10.04(8.99,11.61)	10.03(8.5,11.91)	0.484	-0.536	0.592
Ileucine	12.67(10.96,14.59)	11.91(10.31,13.99)	0.347	-1.708	0.088
TMAO	0.43(0.28,0.66)	0.42(0.27,0.67)	0.325	-0.501	0.616
Betaine	7.78(6.46,9.13)	8.12(6.25,9.17)	0.173	0.109	0.913
C5	0.03(0.02,0.03)	0.02(0.02,0.03)	0.059	-2.266	**0.023**
C18:1-	0.1(0.08,0.11)	0.09(0.08,0.11)	0.049	-0.829	0.407
C10:1	0.13(0.1,0.17)	0.13(0.1,0.19)	0.041	0.357	0.721

Serum concentrations are shown as median and interquartile range(P25, P75)

Abbreviates:LPC16:0:1-palmitoyl-2-hydroxy-sn-glycero-3-phosphocholine,LPC18:0:1-stearoyl-2-hydroxy-sn-glycero-3-phosphocholine,LPC18:1:1-oleoyl-2-hydroxy-sn-glycero-3-phosphocholine,LPC18:2:1-linoleoyl-2-hydroxy-sn-glycero-3-phosphocholine, C2:acetylcarnitine, C5:valeryl-L-carnitine, C10:1:decenoyl-L-carnitine, C18:1:octadecenoyl-L-carnitine, TMAO: trimethylamine oxide

The specific and overlapped metabolic factors for three sarcopenic traits by univariate and multivariate analysis are shown in Fig. 1D.

## Discussion

Sarcopenia-related decreases in muscle mass, strength, and function can result in adverse health outcomes and subsequent loss of independence in older adults. Characterizing the metabolic profile associated with reduced muscle mass and strength in older persons could thus have important translational implications for identifying subjects at high risk of sarcopenia and early preventive strategies and treatments. The present study investigated the associations between metabolites and sarcopenia and its parameters in older Chinese men (mean age 79.2 ± 7.8 years). We used a targeted metabolomics approach and simultaneously analyzed amino acids, carnitine, acylcarnitines, and LPCs to identify specific and overlapped metabolic factors for key sarcopenia traits. The results revealed that C5 level was independently associated with the presence of sarcopenia and was positively correlated with SMI, suggesting that C5 (intermediates and byproducts of BCAA catabolism, a marker of fatty acid oxidation) may act as a potential marker of muscle mass and sarcopenia. Regarding the overlapped metabolites for muscle parameters, ten metabolites were associated with muscle mass, including six (leucine, C8, C10, C12, C14, and LPC18:2) associated with handgrip strength, three of which (C12, C14, and LPC18:2) were also associated with walking speed. Specifically, tryptophan was related positively and glycine was related negatively with handgrip strength, while glutamate was positively associated with gait speed. Additionally, isoleucine, BCAA, and LPC16:0 were positively associated with SMI, respectively. The characterization of the metabolic features of sarcopenia and its components provides a reference of potential mechanism and directions for the management of sarcopenia.

Previous studies have investigated the role of amino acids in sarcopenia. In particular, lower plasma concentrations of the BCAAs leucine and isoleucine were found in older, sarcopenic community-dwelling individuals in Norway, using independent-samples *t*-tests [7, 10]. Conversely, older Japanese people with sarcopenia were characterized by higher concentrations of proline according to logistic regression analysis [11]. Additionally, levels of essential amino acids (EAAs), including lysine, methionine, phenylalanine, and threonine, as well as BCAAs and choline (no specific class was identified), were inversely associated with sarcopenia among 189 older community-dwelling individuals (mean age 73.2 years), according to logistic regression analysis [12]. Aleman-Mateo and colleagues [45] found that seven of the nine EAAs (methionine, lysine, phenylalanine, threonine, and the three BCAAs) were negatively associated with sarcopenia, and were positively associated with muscle mass. In contrast to the specific patterns of circulating amino acids associated with sarcopenia in previous studies, the current results found no significant changes in BCAA, leucine, or isoleucine, which are essential for maintaining muscle strength, but these were associated with SMI by Spearman analysis.

Skeletal muscle mass, which comprises the largest fraction of total body fat-free mass and an essential feature of sarcopenia, has been reported to decline at a rate of 1%–2% per year in persons older than 50 years, resulting in reduced strength and functional capacity [31]. Specific patterns of circulating amino acids, carnitine, and LPC have been associated with muscle mass and quality in older adults. In the cross-sectional Maastricht Sarcopenia study (MaSS), generalized linear modelling and logistic regression showed that lower blood levels of EAAs, total BCAAs, and leucine were associated with lower SMI, whereas no association was found for total amino acids [46]. The Baltimore Longitudinal Study of Aging used targeted metabolomics to reveal lower levels of circulating leucine, isoleucine, tryptophan, serotonin, and methionine in older subjects with poor muscle quality. There was also a decrease in the LPCs acyl (a) C16:1 and C18:1 in patients with lower muscle quality relative to controls, indicating that these metabolites were potential biomarkers for muscle mass decline in older adults [47]. A previous study reported that nine metabolites, including seven BCAAs or BCAA-related metabolites (such as leucine and isoleucine) and isovalerylcarnitine (C5) were positively and significantly associated with both thigh muscle cross-sectional area and fat-free mass index, while one tryptophan-related metabolite (indolepropionate) was negatively related to these parameters. Moreover, negative associations with cross-sectional area were found for two lysophospholipids (1-linoleoylglycerophosphoethanolamine and 2-linoleoylglycerophosphoethanolamine), but no significant associations between lysophosphatidylethanolamines and fat-free mass index were identified [41] in linear regression analysis. Our findings were consistent with prior evidence regarding the associations of C5 and LPC, as well as BCAA and leucine, with SMI. Similar to our study, Murphy and colleagues [26] performed metabolomics analysis of plasma from 319 black men in the Health, Aging and Body Composition (ABC) study, and Pearson’s partial correlations showed that the metabolites most strongly correlated with appendicular skeletal muscle mass included acylcarnitine C5 (positively correlated) and BCAAs. Moreover, Linear regression analysis with sex and leucine as independent variables also showed that leucine concentration was significantly correlated with SMI [10].

Handgrip strength has been proposed as a robust predictor of frailty and sarcopenia. Identifying biomarkers for declining handgrip strength could thus deepen our understanding of the mechanism underpinning sarcopenia. Acylcarnitines are metabolites generated by fatty acid metabolism in the mitochondria, and are dysregulated in multiple disorders affecting the musculature. A previous study showed that baseline levels of short-chain dicarboxylic and hydroxylated acylcarnitines were inversely associated with and significantly explained the variability in 18-month decline in handgrip strength, with the acylcarnitine species C4-OH accounting for most of the variance [48]. In contrast, the current results found no association with short-chain acylcarnitine, while medium- and long-chain acylcarnitine (C8, C10, and especially C12, and C14) were negatively associated with handgrip strength in linear regression analysis. Regarding amino acids, MaSS indicated that lower blood levels of EAAs, total BCAAs, and leucine were associated with lower handgrip strength by linear analysis, whereas no association was found for total amino acids [46]. We found a positive relationship between handgrip strength and leucine according to Spearman’s analysis, and a positive relationship with tryptophan (an aromatic EAA involved in kynurenine metabolism in muscle) and negative relationship with glycine (a non-EAA involved in anti-inflammatory processes, immune function, and anti-oxidation reactions) by both Spearman’s and linear regression analyses.

Gait speed is an important measure of lower extremity physical performance in older adults and is widely used for the consensus definition of frailty and sarcopenia. Previous studies have indicated that walking speed can predict life span [48], and one study has even proposed that walking speed can be used as the sixth vital sign[49]. Toots, et al. have found lower walking speed was significantly associated with an increased risk of all-cause mortality after adjustment for multiple confounders[50]. Physical performance measures, particularly gait speed, have been extensively studied as potential measures for identifying older adults at risk of impaired mobility [24] and cognition, dementia [51, 52], and mortality [53]. Lower blood levels of EAAs, total BCAAs, and leucine were associated with slower gait speed [24]. Among 313 black men in the Health ABC Study (median age 74 years), the metabolites salicylurate and 2-hydroxyglutarate were correlated with gait speed by partial correlation analysis. Metabolites of amino acids and their degradation (indoxy sulfate) were among the 23 metabolites associated with incident impaired mobility by Cox regression models [46]. In the current results, glutamate was positively associated with gait speed by Spearman’s and linear analyses, but there was no association between BCAA or tryptophan and gait speed. Glutamate is a crucial intermediate of muscle energy metabolism and liver-muscle metabolic interchange under both physiologic and pathologic conditions [54]. Perturbations in the circulating glutamate pool may be indicative of skeletal muscle dysfunction and are commonly encountered in age-related chronic conditions and models of muscle atrophy [55]. Our findings of an association between glutamic acid and gait speed support a role of glutamate in presence of sarcopenia.

The most abundant LPC in human plasma is 16:0 followed by 18:2, 18:0/18:1, 20:4, and other minor LPC species. Low plasma LPC 18:2 has previously been shown to predict impaired glucose tolerance, insulin resistance, memory impairment, and lower LPC levels were associated with impaired mitochondrial oxidative capacity of skeletal muscles [16, 56]. Of 148 plasma metabolites (amino acids, biogenic amines, hexoses, glycerophospholipids) measured, LPC 17:0, 18:1, and 18:2 were significantly associated with gait speed, while baseline plasma LPC 18:2 was also an independent predictor of the rate of change of gait speed during subsequent follow-up from the Baltimore Longitudinal Study of Aging [27]. In line with previous studies, we also found that LPC18:2 was positively linked to gait speed by Spearman’s analysis, but not by linear regression analysis. In respect of carnitine and acylcarnitines, they are also involved in mitochondrial transport of fatty acids and are relevant agents for normal mitochondrial function. Medium- and long-chain acylcarnitines are elevated in conditions with vascular inflammation and insulin resistance [57], which alter intracellular energy production and muscle contraction [58]. A small cross-sectional study found significant Spearman correlations between higher plasma concentrations of medium and long-chain acylcarnitines and lower physical performance, measured by the Short Physical Performance Battery total score (including gait speed, balance, and 5STS) [23]. As in our study, the predominant species contributing to this association were the long-chain acylcarnitines (C12 and C14). The negative associations between acylcarnitine levels and gait speed aligned with prior evidence. A case–control study nested in the Seniors-ENRICA cohort of community-dwelling older adults found that higher plasma concentrations of medium- and long-chain acylcarnitines were associated with a higher risk of lower Short Physical Performance Battery [59]. The causes of impaired muscle mass and strength during aging were examined in a previous study, which compared genome-wide transcriptional changes in sarcopenic versus age-matched controls using muscle biopsies from 119 older men. The results provided an integrated molecular profile of human sarcopenia across ethnicities, demonstrating a fundamental role of altered mitochondrial metabolism in the pathological losses of skeletal muscle mass and function in older people [60]. The current findings thus provide further evidence for the contribution of a metabolic-related mitochondrial energy disorder to sarcopenia.

Compared to genes or proteins, metabolites are indeed less stable. They may fluctuate in the short term due to the influence of diet and drugs, but it is generally believed that there is little flux if long-term diet and medication habits on a regular basis, and the metabolites concentration in blood may possibly achieve a detectable stable state [61]. Protein consumption must be equivalent to bodily metabolic demands to prevent the use of skeletal muscle contractile proteins as sources of amino acids in situations of stress and fasting. Hence, an adequate supply of dietary proteins is crucial to maintain body homeostasis and function. Addtionally, essential amino acids are not produced by the human body and must be acquired through their extraction from dietary protein [62]. Insufficient protein intake to satisfy daily requirements leads to negative protein balance and results in skeletal muscle atrophy and functional decline [63]. One study have showed significantly lower absolute intake of protein when compared with non-sarcopenic and non-frail older adult [10]. A higher protein intake has been recommended in older vs. younger adults to maximally stimulate postprandial muscle protein synthesis and prevent sarcopenia. The influence of dietary protein intake on the metabolome is not well understood, although it can be hypothesized that potential health effects are mediated by changes in specific metabolites. In addition to dietary intake, the body can also maintain its homeostasis by regulating the catabolism of amino acids. At present, it is not clear whether supplemental BCAA will definitely cause the increase of circulating BCAA level, at least the correlation is not as strong as expected. For example, the American Nurses' Health Study (NHS II) found that dietary intake of BCAA was only weakly positively correlated with circulating BCAA level [64]. In this study, overnight fasting blood samples were collected uniformly to increase comparability. Unfortunately, we have no standardised information to report on daily protein intake level. The level flux of metabolites may have occurred over time, which needs to be further investigated.

The present study had several strengths. First, we identified the metabolic signature of sarcopenia by measuring an multiple array of acylcarnitines and LPCs as well as amino acids. Furthermore, we investigated the independent associations of these metabolic factors with the presence of sarcopenia and its components in older men. Second, we considered both muscle mass and muscle strength, which reflect important pathophysiological aspects of sarcopenia, and identified both specific and shared metabolites related to these sarcopenic components, providing important information to further our understanding of the pathogenesis of sarcopenia. Our findings suggests that the three classes of metabolites, especially acylcarnitines and LPCs, should be further investigated to elucidate the clinical relevance and potential biomarkers in sarcopenic older adults, which might have a key role to foster the adherence to nutritional recommendations. However, some limitations should be considered when interpreting the study findings. First, the causal relationships between the identified metabolites and sarcopenic traits could not be inferred because of the cross-sectional study design. Future follow-up studies allow for the conclusions to be drawn regarding possible cause-effect relationships. Second, the relatively small sample size may limit the statistical power of the study to identify sarcopenia-trait-related metabolites. Moreover, the study analyzed a convenience sample of older men, rather than being a population-based study, and the findings cannot necessarily be generalized to other study populations due to social and cultural differences. Third, although the latest AWGS2 and EWGSOP2both suggest using BIA measurement of ASM to assess muscle mass, the use of BIA has some flaw as the gold standard for muscle mass measurement is computed tomography (CT), magnetic resonance (MRI).Fourth, we cannot exclude the possibility that there may have been some unmeasured, confounding factors, such as eating habits and lifestyle, after adjusting for the available covariates. The lack of assessment of dietary protein-related parameters could not provide a more comprehensive appraisal of this relationship. Whether dietary intake of protein over time leads to the change of blood concentration of acylcarnitines, LPCs and amino acids, in particular, needs to be further investigated.

## Conclusions

In summary, the current targeted LC–MS/MS-based metabolic analysis of serum samples provides a reference for the metabolic features associated with sarcopenia and its main components. We explored the differential metabolites between sarcopenia and non-sarcopenia, and revealed that the main metabolic alterations in sarcopenia were related to acylcarnitines and LPC metabolites as well as amino acids. Characterizing the metabolic profile associated with sarcopenia and its muscle parameters could have important translational implications for potential mechanism and management of sarcopenia in older persons. Our findings suggest that specific LPC and acylcarnitines ametabolites may contribute to sarcopenia development and maintaining optimizing their concentrations may be beneficial to muscle-related mitochondrial energy in the sarcopenic older adults. However, further studies are needed to confirm our findings and clarify the potential roles of the metabolites associated with sarcopenia.

## Supplementary Information

**Additional file 1.** **Suppl.Table 1. **Liner regression analysis for associations of serum metabolites with gait speed. **Suppl.Table 2. **Liner regression analysis for associations of serum metabolites with handgrip strength. **Suppl.Table 3. **Liner regression analysis for associations of serum metabolites with SMI. **Figure 1.** Validation of OPLS-DA analysis.** (A)** Permutation tests of OPLS-DA model analysis for sarcopenia versus nonsarcopenia groups. **(B) **The area under the curve (AUC) for separation of sarcopenia and nonsarcopenia groups.

## Data Availability

The datasets used and/or analysed during the current study are available from the corresponding author on reasonable request.
