# Supplementary material for: Specific lysophosphatidylcholine and acylcarnitine related to sarcopenia and its components in older men
Source: BMC Geriatr. 2022 Mar 25;22:249. doi: 10.1186/s12877-022-02953-4 (PMC8957177; doi:10.1186/s12877-022-02953-4)
Supplement: Supplementary file 1 — Additional file 1. Suppl.Table 1. Liner regression analysis for associations of serum metabolites with gait speed. Suppl.Table 2. Liner regression analysis for associations of serum metabolites with handgrip strength. Suppl.Table 3. Liner regression analysis for associations of serum metabolites with SMI. Figure 1. Validation of OPLS-DA analysis. (A) Permutation tests of OPLS-DA model analysis for sarcopenia versus nonsarcopenia groups. (B) The area under the curve (AUC) for separation of sarcopenia and nonsarcopenia groups. [file 12877_2022_2953_MOESM1_ESM.docx]

**Suppl.Table 1 Liner regression analysis for associations of serum metabolites with gait speed**

| variables | MODEL1 | |  | |  | | |  | MODEL2 | | |  | |
| --- | --- | --- | --- | --- | --- | --- | --- | --- | --- | --- | --- | --- | --- |
|  | *β* | *SE* | | *P* | |  | *β* | | | *SE* | *P* | |  |
| A1 | 0.0017 | 0.00404 | | 0.6751 | |  | -0.0012 | | | 0.00419 | 0.7673 | |  |
| A2 | 0.00763 | 0.01079 | | 0.4803 | |  | -0.0010 | | | 0.01134 | 0.9288 | |  |
| A3 | 0.00690 | 0.00632 | | 0.2760 | |  | 0.00249 | | | 0.0066 | 0.7062 | |  |
| A4 | 0.00651 | 0.0087 | | 0.4539 | |  | 0.00463 | | | 0.0088 | 0.5976 | |  |
| A6 | 0.01527 | 0.00892 | | 0.0880 | |  | 0.0164 | | | 0.0090 | 0.0687 | |  |
| A7 | -0.0001 | 0.00372 | | 0.9762 | |  | -0.0016 | | | 0.0037 | 0.6747 | |  |
| A8 | 0.01065 | 0.0053 | | 0.0443 | |  | 0.01040 | | | 0.0053 | 0.0488 | |  |
| A9 | -0.0014 | 0.0013 | | 0.2601 | |  | -0.0018 | | | 0.0013 | 0.1559 | |  |
| A11 | -0.0740 | 0.0572 | | 0.1972 | |  | -0.0910 | | | 0.0572 | 0.1130 | |  |
| A12 | 0.0015 | 0.0021 | | 0.4680 | |  | -0.0001 | | | 0.0022 | 0.9623 | |  |
| C2 | -0.0732 | 0.0362 | | 0.0443 | |  | -0.0697 | | | 0.0366 | 0.0584 | |  |
| C5 | 2.4953 | 3.0638 | | 0.4162 | |  | 1.1260 | | | 3.1400 | 0.7202 | |  |
| C8 | -0.2946 | 0.5832 | | 0.6139 | |  | -0.3998 | | | 0.6030 | 0.5081 | |  |
| C10 | -0.3029 | 0.4593 | | 0.5102 | |  | -0.4336 | | | 0.4753 | 0.3626 | |  |
| C12 | -3.2326 | 1.9922 | | 0.1060 | |  | -4.3367 | | | 2.0207 | 0.0329 | |  |
| C14 | -13.1373 | 6.1777 | | 0.0345 | |  | -16.7394 | | | 6.2194 | 0.0076 | |  |
| C16 | -1.2876 | 2.1475 | | 0.5494 | |  | -2.3072 | | | 2.1656 | 0.2878 | |  |
| L1 | 0.0015 | 0.0009 | | 0.0832 | |  | 0.0015 | | | 0.0008 | 0.0797 | |  |
| L4 | 0.0033 | 0.0024 | | 0.1765 | |  | 0.0036 | | | 0.0024 | 0.1306 | |  |

Model 1: age, body mass index, smoking status, alcohol drinking, malnutrition and physical activity, Model 2: further controlled for polypharmacy and comorbidities .

Abbreviates: SMI: skeletal muscle index, Glu: glutamate, Gln: glutamine, BCAA: branched-chain-amino acids, C2:acetylcarnitine, C5:valeryl-L-carnitine,C8:octanoyl-L-carnitine,C10:decanoyl-L-carnitin,C12:dodecanoy-L-carnitine,C14:tetradecanoyl-L-car-nitine,C16:hexadec-C16anoyl-L-carnitine, LPC16:0:1-palmitoyl-2-hydroxy-sn-glycero-3-phosphocholine, LPC18:2: 1-linoleoyl-2-hydroxy-sn-glycero-3-phosphocholine

**Suppl.Table 2 Liner regression analysis for associations of serum metabolites with handgrip strength**

| variables | MODEL1 | |  | |  | | |  | MODEL2 | | |  | |
| --- | --- | --- | --- | --- | --- | --- | --- | --- | --- | --- | --- | --- | --- |
|  | *β* | *SE* | | *P* | |  | *β* | | | *SE* | *P* | |  |
| A1 | -0.0289 | 0.0592 | | 0.6264 | |  | -0.0302 | | | 0.0622 | 0.6274 | |  |
| A2 | -0.0879 | 0.15830 | | 0.5792 | |  | -0.0884 | | | 0.1680 | 0.5992 | |  |
| A3 | 0.0054 | 0.0929 | | 0.9540 | |  | 0.01254 | | | 0.0977 | 0.8979 | |  |
| A4 | 0.14601 | 0.1271 | | 0.2518 | |  | 0.13643 | | | 0.1298 | 0.2942 | |  |
| A6 | 0.3728 | 0.12933 | | 0.0043 | |  | 0.3621 | | | 0.13221 | 0.0066 | |  |
| A7 | 0.00158 | 0.05452 | | 0.9769 | |  | 0.0087 | | | 0.0555 | 0.8755 | |  |
| A8 | 0.04682 | 0.0778 | | 0.5481 | |  | 0.0408 | | | 0.0785 | 0.6036 | |  |
| A9 | -0.0408 | 0.0183 | | 0.0271 | |  | -0.0386 | | | 0.0190 | 0.0429 | |  |
| A11 | -0.6478 | 0.8416 | | 0.4423 | |  | -0.5546 | | | 0.8524 | 0.5159 | |  |
| A12 | -0.01082 | 0.0312 | | 0.7291 | |  | -0.0105 | | | 0.0330 | 0.7507 | |  |
| C2 | -0.5302 | 0.5345 | | 0.3222 | |  | -0.4727 | | | 0.5466 | 0.3880 | |  |
| C5 | 7.9739 | 44.9990 | | 0.8595 | |  | 18.8662 | | | 46.5529 | 0.6856 | |  |
| C8 | -11.2777 | 8.5277 | | 0.1873 | |  | -14.4783 | | | 8.8996 | 0.1051 | |  |
| C10 | -9.9111 | 6.7124 | | 0.1411 | |  | -12.4458 | | | 7.0136 | 0.0773 | |  |
| C12 | -56.2589 | 29.1559 | | 0.0548 | |  | -61.3959 | | | 29.9864 | 0.0417 | |  |
| C14 | -219.5502 | 90.3594 | | 0.0158 | |  | -228.4 | | | 92.4307 | 0.0142 | |  |
| C16 | -8.6282 | 31.5181 | | 0.7845 | |  | -8.9734 | | | 32.1811 | 0.7806 | |  |
| L1 | 0.0040 | 0.0126 | | 0.7534 | |  | 0.0029 | | | 0.0127 | 0.8183 | |  |
| L4 | 0.0638 | 0.0352 | | 0.0713 | |  | 0.0605 | | | 0.0356 | 0.0904 | |  |

Model 1: age, body mass index, smoking status, alcohol drinking, malnutrition and physical activity, Model 2: further controlled for polypharmacy and comorbidities .

Abbreviates: SMI: skeletal muscle index, Glu: glutamate, Gln: glutamine, BCAA: branched-chain-amino acids, C2:acetylcarnitine, C5:valeryl-L-carnitine,C8:octanoyl-L-carnitine,C10:decanoyl-L-carnitin,C12:dodecanoy-L-carnitine,C14:tetradecanoyl-L-car-nitine,C16:hexadec-C16anoyl-L-carnitine, LPC16:0:1-palmitoyl-2-hydroxy-sn-glycero-3-phosphocholine, LPC18:2: 1-linoleoyl-2-hydroxy-sn-glycero-3-phosphocholine

**Suppl.Table 3 Liner regression analysis for associations of serum metabolites with SMI**

| variables | MODEL1 | |  | |  | | |  | MODEL2 | | |  | |  |
| --- | --- | --- | --- | --- | --- | --- | --- | --- | --- | --- | --- | --- | --- | --- |
|  | *β* | *SE* | | *P* | |  | *β* | | | *SE* | *P* | |  | |
| A1 | -0.0142 | 0.0142 | | 0.3170 | |  | -0.0093 | | | 0.0147 | 0.5264 | |  | |
| A2 | -0.0223 | 0.0380 | | 0.5577 | |  | -0.0097 | | | 0.0397 | 0.8065 | |  | |
| A3 | -0.0093 | 0.0223 | | 0.677 | |  | -0.0030 | | | 0.0231 | 0.8967 | |  | |
| A4 | -0.0477 | 0.0304 | | 0.119 | |  | -0.0332 | | | 0.0306 | 0.2797 | |  | |
| A6 | -0.0365 | 0.0315 | | 0.2482 | |  | -0.02099 | | | 0.0317 | 0.5087 | |  | |
| A7 | -0.0110 | 0.0131 | | 0.3988 | |  | -0.01316 | | | 0.0131 | 0.3157 | |  | |
| A8 | -0.0018 | 0.0187 | | 0.9244 | |  | 0.00394 | | | 0.0186 | 0.8319 | |  | |
| A9 | -0.0032 | 0.0044 | | 0.4702 | |  | -0.0062 | | | 0.0045 | 0.1709 | |  | |
| A11 | -0.1112 | 0.2022 | | 0.5829 | |  | -0.1556 | | | 0.2015 | 0.4407 | |  | |
| A12 | -0.0059 | 0.0075 | | 0.4339 | |  | -0.0034 | | | 0.0078 | 0.6683 | |  | |
| C2 | 0.02550 | 0.1286 | | 0.8430 | |  | -0.0474 | | | 0.1294 | 0.7141 | |  | |
| C5 | 18.2442 | 10.7398 | | 0.0907 | |  | 15.4136 | | | 10.964 | 0.1611 | |  | |
| C8 | -1.2738 | 2.0532 | | 0.5356 | |  | 0.4371 | | | 2.1156 | 0.8365 | |  | |
| C10 | -1.2914 | 1.6168 | | 0.4252 | |  | 0.0445 | | | 1.6692 | 0.9788 | |  | |
| C12 | -4.9331 | 7.0471 | | 0.4846 | |  | -1.4993 | | | 7.1513 | 0.8341 | |  | |
| C14 | -25.2282 | 21.8999 | | 0.2505 | |  | -19.4888 | | | 22.096 | 0.3787 | |  | |
| C16 | -3.6715 | 7.5646 | | 0.6279 | |  | -1.4844 | | | 7.6090 | 0.8455 | |  | |
| L1 | 0.0052 | 0.0030 | | 0.0866 | |  | 0.0060 | | | 0.0030 | 0.0438 | |  | |
| L4 | 0.0097 | 0.0085 | | 0.2542 | |  | 0.0122 | | | 0.0084 | 0.1482 | |  | |

Model 1: age, body mass index, smoking status, alcohol drinking, malnutrition and physical activity, Model 2: further controlled for polypharmacy and comorbidities.

Abbreviates: SMI: skeletal muscle index, Glu: glutamate, Gln: glutamine, BCAA: branched-chain-amino acids, C2:acetylcarnitine, C5:valeryl-L-carnitine,C8:octanoyl-L-carnitine,C10:decanoyl-L-carnitin,C12:dodecanoy-L-carnitine,C14:tetradecanoyl-L-car-nitine,C16:hexadec-C16anoyl-L-carnitine, LPC16:0:1-palmitoyl-2-hydroxy-sn-glycero-3-phosphocholine, LPC18:2: 1-linoleoyl-2-hydroxy-sn-glycero-3-phosphocholine

**Suppl. Figure1**


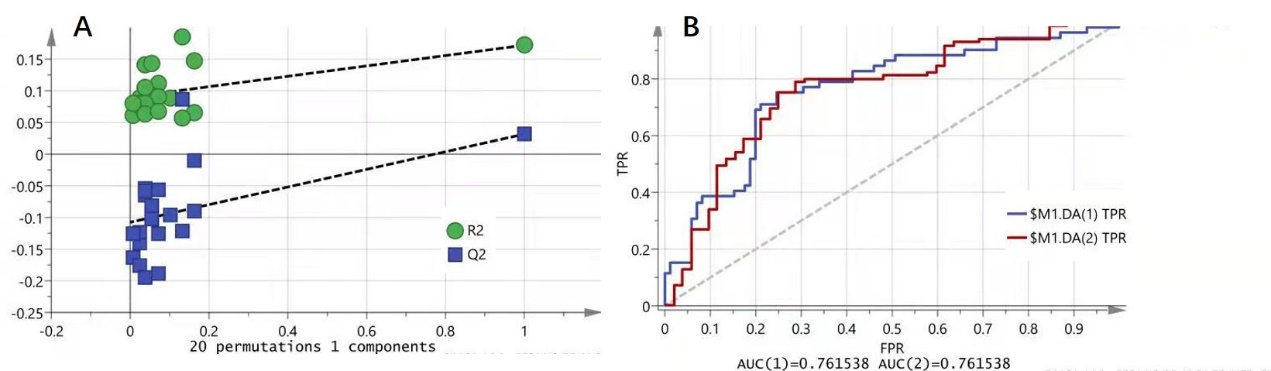


**FIGURE 1** Validation of OPLS-DA analysis. **(A)** Permutation tests of OPLS-DA model analysis for sarcopenia versus nonsarcopenia groups. **(B)** The area under the curve (AUC) for separation of sarcopenia and nonsarcopenia groups.
